# Eudaimonic Well-Being and Coping with Stress in University Students: The Mediating/Moderating Role of Self-Efficacy

**DOI:** 10.3390/ijerph16010048

**Published:** 2018-12-25

**Authors:** Carlos Freire, María del Mar Ferradás, José Carlos Núñez, Antonio Valle, Guillermo Vallejo

**Affiliations:** 1 Department of Psychology, University of A Coruña, A Coruña, 15071 Galicia, Spain; mar.ferradasc@udc.es (M.M.F.); vallar@udc.es (A.V.); 2 Faculty of Psychology, University of Oviedo, Oviedo, 33003 Asturias, Spain; jcarlosn@uniovi.es (J.C.N.); gvallejo@uniovi.es (G.V.)

**Keywords:** eudaimonic well-being, self-efficacy, coping strategies, stress, university students

## Abstract

The importance of personal psychological resources in preventing academic stress has enjoyed little attention to date, despite the high rates of stress that exist among university students. This article analyzes the effect of eudaimonic well-being on the use of adaptive strategies for coping with academic stress. Moreover, it analyzes the role of self-efficacy as a mediator and moderator of this relationship. In the mediation model, gender is included as a co-variable; in the moderation model, gender is included as a moderator. A total of 1402 university students participated in the study. The data were gathered through validated self-report instruments. The mediation analyses were performed using the PROCESS module of the statistical package, SPSS. The moderating effects of self-efficacy and gender were analyzed through hierarchical regression analysis. The results indicate that self-efficacy partially mediates but does not moderate the relationship between eudaimonic well-being and adaptive coping strategies. This finding reveals the benefits of using these two personal resources to enhance effective coping with academic stress while attending university.

## 1. Introduction

Researchers focusing on academic stress attest to its high prevalence during students’ time at university [1,2]. In their daily lives, university students face worries such as adapting to a new environment, academic performance, overwork, or future employment [3,4,5], as well as others that are social and financial in nature [6]. These factors are often perceived by students as highly stressful and may cause significant damage to their performance [7] as well as their health [6,8]. 

The short- and long-term impact of these potential stressors depends in large part on the capacity of individuals to face them in an adaptive manner [9]. Therefore, assuming that an individual’s resources play an important role in effective stress management, hand in hand with positive psychology, there is growing scientific interest in the personal strengths that contribute significantly to achieving optimal positive functioning in humans [10,11]. Personal strengths become real psychological resources that allow individuals to not only reduce pathological states [12], but also to stimulate their personal development [13]. 

The prime example of this recent research perspective is eudaimonic well-being, which is focused on the blossoming of capacities and individual potential as a route to achieving full psychological functioning. Ryff [14] proposes that human eudaimonic well-being comprises six personal resources: self-acceptance, positive relationships with other people, autonomy, environmental mastery, life purpose, and personal growth. 

### 1.1. Eudaimonic Well-Being and Coping with Stress

To date, few studies have analyzed the role of eudaimonic well-being (taken as a global construct) as an antecedent of processes of coping with academic stress. Some articles regarding high-school students [15,16] have demonstrated that high levels of eudaimonic well-being favor the adoption of highly adaptive coping strategies, such as positive reevaluation of problems, engagement with tasks or the search for help for instrumental and emotional purposes. In contrast, students with low levels of eudaimonic well-being turned more frequently to maladaptive coping strategies such as blaming oneself for problems, ignoring them, or escaping through fantasizing thoughts. Along these same lines, Freire, Ferradás, Valle, Núñez, and Vallejo [17] found that university students with high eudaimonic well-being (as opposed to those with low eudaimonic well-being) turned to a greater extent to strategies of positive reevaluation, seeking out support, and planning. 

More research has focused on analyzing, in an isolated way, the role played by some variables associated with some components of eudaimonic well-being, such as self-esteem [18] or purpose in life [19], in coping with stress. Self-efficacy stands out prominently among the most frequently studied aspects linked to eudaimonic well-being.

### 1.2. Self-Efficacy and Coping with Stress

Self-efficacy has been characterized as an individual’s belief regarding his or her own capacity to achieve a desired objective or standard [20]. Self-efficacy consists of a universal psychological need [21], linked to eudaimonic well-being [22], that regulates an individual’s cognitions, motivations, emotions and decisions [23,24]. In this sense, self-efficacy plays a relevant role in processes of coping with stress [25], having a particular influence on the evaluation of stressors [26], as well as the selection and execution of strategies used to face them [27]. Thus, those individuals with high self-efficacy tend to evaluate potentially stressful situations as challenges rather than as threats [23,28]. This tendency makes them more inclined, compared to individuals with low self-efficacy, to use highly adaptive coping strategies [29,30,31]. Therefore, self-efficacy comprises an important personal resource in favor of preventing academic stress in university students [32,33], facilitating adaptive adjustment to this formative phase [34]. 

### 1.3. About this Study

The studies reviewed above demonstrate that both eudaimonic well-being and self-efficacy independently favor adaptive coping with academic stress. However, both personal resources could have a joint impact on coping strategies used by university students. The Conservation of Resources (COR) theory [35,36] proposes that individuals who possess high personal resources demonstrate high motivation to acquire, maintain and promote new resources, and this spiral of positive gains would report adaptive personal results in the long term. In accordance with this theory, some articles [37,38] state that psychological resources linked to personal development promote high self-efficacy among university students. Therefore, it is possible that individuals with high eudaimonic well-being may have a greater tendency toward developing high self-efficacy, and both resources jointly favor more adaptive coping with stress.

Based on this approach, the first objective of this study is to analyze the role of self-efficacy as a mediating variable in the relationship between eudaimonic well-being and the use of adaptive coping strategies in the academic context. Self-efficacy would act as a mediating variable if the effect of eudaimonic well-being on coping strategies was conditioned, at least partially, by the effect of self-efficacy on these strategies. 

Some studies among university students have demonstrated the mediating effect of self-efficacy between some psychological resources, such as positive emotions or mindfulness, and the adoption of strategies such as engagement with academic tasks or positive re-evaluation of problems [39,40]. Along these same lines, Loton and Waters [41] observed that high-school students’ perceptions about the degree to which their parents encouraged them to use their personal strengths exerted an indirect positive effect, through self-efficacy, on reducing stress. Therefore, the main contribution of this study is the inclusion of the eudaimonic well-being construct, as it constitutes a more global and representative example of the functioning of positive psychology [14] than those contributed by previous research. Drawing on the works cited above, our hypothesis in relation to this first objective is that the eudaimonic well-being of university students will have a significant positive effect, both direct and indirect (through self-efficacy), on adaptive coping strategies.

Similarly, some studies [20,42] argue that self-efficacy adopts a relevant role in work-related stress, playing a moderating role. Therefore, assuming that, from a psychological point of view, academic assignments can be considered “work” [43], it is plausible that self-efficacy not only mediates but also moderates the relationship between eudaimonic well-being and adaptive coping strategies. In this sense, COR theory maintains that while the real or potential loss of personal resources is stressful, individuals can employ other resources to compensate for this loss [35]. From this perspective, although lacking eudaimonic well-being constitutes a risk factor for experiencing depressive emotional states [44], it is possible that self-efficacy constitutes a psychological resource capable of moderating the harmful effects caused by the absence of eudaimonic well-being on adaptive coping with stress.

Previous research offers only indirect evidence regarding this issue. It has been observed in work environments that individuals who experience symptoms of poor psychological adjustment (in the form of role overload and conflict, emotional dissonance, low perceived autonomy, or low satisfaction) with high levels of self-efficacy tend to evaluate demands as challenges [28] and engage in functional coping strategies [45], which leads them to experience low levels of stress [46]. 

Taking these contributions as a reference, this article examines not only the mediating hypothesis but also the potential moderating effect of self-efficacy on the relationship between eudaimonic well-being and the adoption of adaptive coping strategies. This objective, unique to date, implies that the relationship between eudaimonic well-being and the use of adaptive coping strategies varies based on levels of self-efficacy. Specifically, it is expected that, among students with low levels of eudaimonic well-being, students who demonstrate greater self-efficacy will use significantly more adaptive coping strategies than students with lower self-efficacy. 

Finally, it should be noted that previous research indicates that men and women face academic stress in university differently [47,48]. As a result, in the study of the hypothesis of mediation and/or moderation, gender will be included in the corresponding model of analysis.

## 2. Materials and Methods

### 2.1. Participants

This research was carried out with students from the University of A Coruña, which is a Spanish university with an enrollment of 17,227 undergraduate students. To achieve the most representative sample of the university population (undergraduates) possible, all degree programs offered at the university were invited to participate in the study. The following programs accepted the invitation: Educational sciences degrees (early childhood education, elementary education, physical education teaching, listening and language teaching, social education, speech therapy and psychopedagogy), health sciences programs (physical therapy, nursing and physical education and sports sciences), technical careers (architecture, technical architecture and engineering of roads, canals and ports), and programs in the legal/social arena (law and sociology). Each of these degrees had four or five cohorts, and each cohort had several groups of students. A group was chosen randomly from each of the cohorts. In this way, an initial sample of 1461 students (8.48%) was obtained. The initial review of the data revealed that 18 cases (1.23%) presented a high number of missing values in the questionnaires, and thus, they were excluded from the study. For the rest of the participants who presented a smaller number of missing values (2.81%), these values were treated statistically using the full information maximum likelihood (FIML) estimation procedure in the Mplus program, version 7.11 [49].

The final sample of participants totaled 1402 students (*M*_age_ = 21.11; *SD* = 3.29); 68.8% (*n* = 964) women and 31.2% (*n* = 438) men. With regard to degree programs, 36.6% (*n* = 513) were pursuing a degree in educational sciences, 18% (*n* = 252) were pursuing a degree in health sciences, 27.3% (*n* = 384) were pursuing a technical career and 18.1% (*n* = 253) were pursuing a legal/social degree. Meanwhile, 28.25% of participants (*n* = 396) were in their first year of university, 23.68% (*n* = 332) were in their second year, 25.53% (*n* = 358) were in their third year, 13.84% (*n* = 194) were in their fourth year, and 8.7% (*n* = 122) were in their fifth year.

### 2.2. Instruments

Eudaimonic well-being. Eudaimonic well-being has been measured by Ryff’s Scales of Psychological Well-Being. Although this tool has received broad empirical support in different cultural contexts [50,51,52,53,54,55,56] and is thus being widely used, some studies have questioned its psychometric properties [57,58,59]. Specifically, the validity of the six-factor structure (i.e., self-acceptance, positive relationships with others, autonomy, environmental mastery, purpose in life, and personal growth) originally proposed by Ryff [60] has been discussed, and there is no full consensus on what and how many factors comprise the eudaimonic well-being construct. In this regard, several studies with Spanish populations [17,61,62] have found empirical and theoretical support for a model that comprises four factors: self-acceptance, environmental mastery, purpose in life, and personal growth. These four personal resources have been considered by several scholars [63,64,65] as the nucleus of eudaimonic well-being. Therefore, the validated Spanish version of Ryff’s Scales of Psychological Well-Being for university students was used [61]. This instrument comprises 18 items that address the four aforementioned core dimensions of eudaimonic well-being: Self-acceptance (three items; e.g., “In general, I feel secure and positive about myself”); personal growth (four items; e.g., “I have the sensation that over time, I have developed a lot as a person”); environmental mastery (five items; e.g., “In general, I feel that I am responsible for my situation in life”); and purpose in life (six items; e.g., “I am clear about the direction and objective of my life”). In this study, the internal consistency of the factors was as follows: self-acceptance (α = 0.79), personal growth (α = 0.63), environmental mastery (α = 0.64) and purpose in life (α = 0.76). Participants’ responses were measured through a 5-point Likert scale, with 1 being strongly disagree and 5 strongly agree. 

Self-efficacy. The General Self-Efficacy Scale was used [66]. This instrument comprises 10 items (e.g., “I can solve difficult problems if I try hard enough”), to which participants responded through a 5-point Likert scale (1 = never—5 = always). The reliability of the scale in our study was α = 0.92. 

Adaptive coping strategies. The Coping Scale from the Academic Stress Questionnaire was used [67]. This scale includes 23 items that evaluate three highly adaptive coping strategies in the academic context [9]: Positive reevaluation (10 items; e.g., “When I face a problematic situation, I forget about the disagreeable aspects and highlight the positive”); seeking support (seven items; e.g., “When I face a problematic situation, I ask for advice from a family member or a friend I appreciate”); and planning (six items; e.g., “When I face a difficult situation, I make a list of the tasks I must do, I do them one by one, and I do not move on to the next until I have completed the previous one”). This structure demonstrated adequate reliability in our study: α = 0.87 (positive reevaluation), 0.91 (seeking support) and 0.83 (planning). Participants’ responses were recorded on a 5-point Likert scale (1 = never—5 = always).

### 2.3. Procedure

The study was conducted according to the guidelines of the Ethics Committee at the University of A Coruña (ethical code: 03/04/2018) [68], with written informed consent from all participants, as established in the Helsinki Declaration. Before the study began, participants were informed of the research objectives and the procedure to be followed to complete the test. Participation in the study was voluntary, and students were guaranteed that their answers would be anonymous and confidential and that the results would be used exclusively for research purposes. The data were gathered in each of the schools at which participants were enrolled in university studies, within classes and during academic hours. The questionnaires were applied by trained personnel in a single session with no time limit. Students who declined to participate in the study left the classroom until after the test was finished. 

### 2.4. Data Analysis

**Figure 1:**
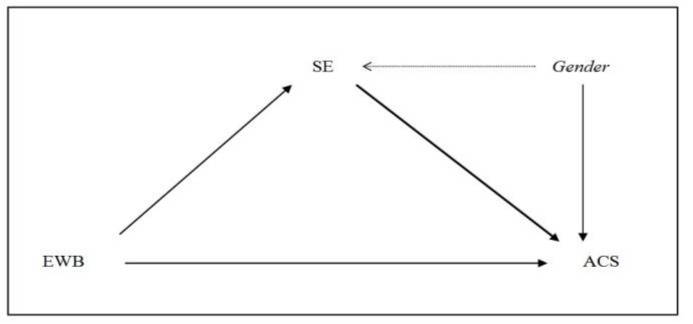
Mediational model. EWB = Eudaimonic Well-Being; SE = Self-Efficacy; ACS = Adaptive Coping Strategies.

The data were analyzed in three phases. First, in the preliminary analysis section, analysis of the correlation matrix and distribution of the variables was conducted, as well as of gender differences in eudaimonic well-being (EWB), self-efficacy (SE) and adaptive coping strategies (ACS). In the second phase, analysis of the mediating role of SE in the effect of EWB on the use of ACS was performed. Figure 1 presents the mediational scheme to be contrasted: It is hypothesized that SE mediates the effect of EWB on the use of ACS (the variable of gender was included to statistically control for its effects). 

Finally, an analysis of the moderation of SE and gender on the effect of EWB in the use of ACS was carried out. In causal terms, while the mediators are positioned between the independent and dependent variables, the moderators always function as independent variables. According to Baron and Kenny [69] (p. 1176), “whereas moderator variables specify when certain effects will hold, mediators speak to how or why such effects occur.”

In a moderation model, in our study, it is expected that the variable of SE will moderate or condition the relationship between EWB and ACS, such that the relationship between well-being and the response changes based on the value assumed by SE. Due to the metric status of predictors of EWB and SE, we will address the moderating role of SE using a regression model. If the existence of moderation is proven, we will graphically represent the regression slopes of the relationship between the predictor and the response for specific points of the moderator.

**Figure 2:**
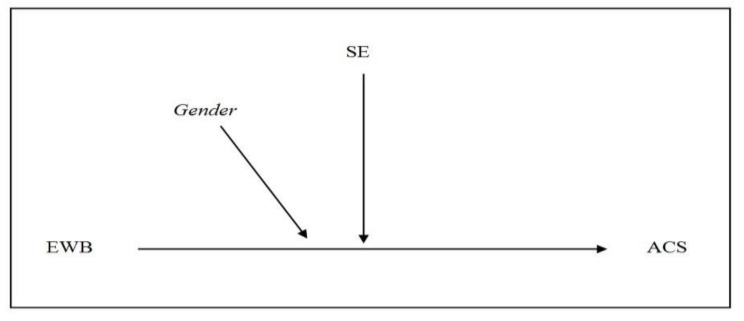
Moderation model. EWB = Eudaimonic Well-Being; SE = Self-Efficacy; ACS = Adaptive Coping Strategies.

Statistically, the effect of moderation is defined as the interaction between the predictor and the moderator, and its analysis involves using the hierarchical regression procedure to test two alternative models. The first incorporates EWB and SE as predictors (Model 1), and the second also includes an interaction between the two (Model 2). To attempt to control the increase in the degree of collinearity in the resulting model, which is a direct consequence of including a product variable, we will mean center the predictors. As in the mediation analysis, in both models, the possible influence of the variable of gender is controlled, as well as its interaction with the predictors of interest (see Figure 2).

The mediation analyses were carried out with the PROCESS model of the statistical package SPSS (version 22.0, IBM Corp., Armonk, NY, USA) [70]. The effect sizes are calculated using Cohen’s d [71]: *d* < 0.20 = minimum effect size; *d* > 0.20 < 0.50 = small effect size; *d* > 0.50 < 0.80 = medium effect size; *d* > 0.80 = large effect size.

## 3. Results

### 3.1. Preliminary Analyses

Table 1 provides the descriptive statistics for the variables and the Pearson correlation matrix. In general, the data indicate that the greater the EWB, the greater the use of ACS and greater SE, and vice versa. The level of significance of the correlations between the three variables was large: *d* (*r*EWB-SE) = 1.256; *d* (*r*EWB-ACS) = 1.320; *d* (*r*ACS-SE) = 1.405. Considering the variable of gender, it was found that women demonstrate less EWB than men (*F* (1.1400) = 4.85; *p* < 0.05; *d* = 0.11), less SE (*F* (1.1400) = 97.75; *p* < 0.001; *d* = 0.53) and less use of ACS (*F* (1.1400) = 5.21; *p* < 0.05; *d* = 0.13). However, the effect size is small, except in the case of SE, which is medium. Finally, according to the asymmetry and kurtosis values, univariate normality is observed in the three variables of interest.

### 3.2. Mediation Analysis

**Figure 3:**
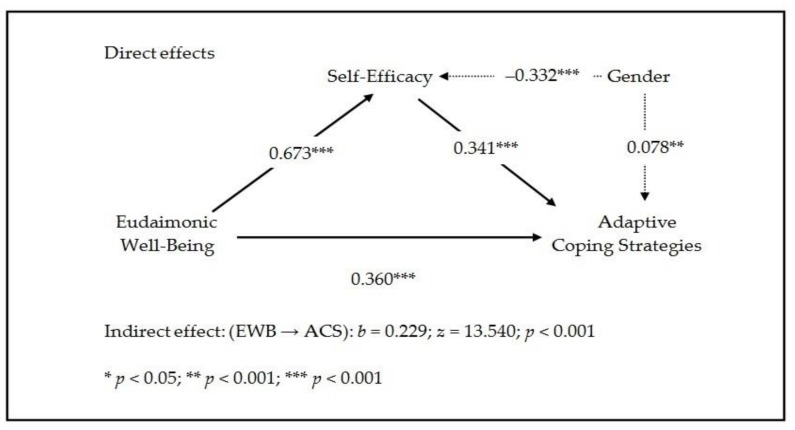
Graphical representation of the results of the mediation model [Gender (Male = 1; Female = 2)].

The results of the mediation analysis are provided in Table 2 and Figure 3. In general terms, the data obtained confirm the initial hypothesis that SE partially mediates the effect of EWB on the use of ACS. As demonstrated in Table 2, EWB has a significant, strong and positive effect on SE (*b* = 0.673; *p* < 0.001; *d* = 1.64), and SE also has a significant, strong and positive effect on the use of ACS (*b* = 0.341; *p* < 0.001; *d* = 0.98). Thus, although EWB has a significant, positive direct effect on the use of ACS (*b* = 0.360; *p* < 0.001; *d* = 0.80), it also has a large, significant, positive indirect effect on the use of ACS through SE (*b* = 0.229; *z* = 13.540; *p* < 0.001; *d* = 0.79). The total effect of EWB on the use of ACS is large, statistically significant and positive (*b* = 0.589; *p* < 0.001; *d* = 1.75).

Based on the data in Table 2, it is also concluded that including the variable of gender in the simple mediation model was correct. Specifically, it was observed that gender had a significant effect, both on SE (upon attempting to determine the effect of EWB on SE) (*b* = −0.332; *p* < 0.001; *d* = 0.57) and on the use of ACS (upon determining whether SE influenced the use of ACS) (*b* = 0.078; *p* < 0.01; *d* = 0.16), but it was not found to have a significant effect when analyzing the direct effect of EWB on the use of ACS (*b* = −0.035; *p* > 0.05).

### 3.3. Moderation Analysis

The results in Table 3 indicate that SE does not alter the relationship between EWB and ACS (*b* = 0.051, *p* > 0.05; *d* = 0.09). Therefore, it can be stated that SE mediates but does not moderate the relationship between EWB and ACS. When a mediation model and a moderation model are adjusted for the same variables, the researcher must continue modeling efforts by seeking more appropriate models to explain this ambiguity [72]. The results demonstrate that ACS were positively associated with EWB (*b* = 0.195, *p* < 0.01; *d* = 0.12) and SE (*b* = 0.338, *p* < 0.001; *d* = 0.97). In other words, an increase of one unit in the predictors of well-being and SE produced a significant increase of 0.195 and 0.338 units, respectively, in the scale of the response variable, with the remaining predictors remaining constant. Consequently, the simplest model was accepted, that is, Model 1, and it was concluded that SE acted as a mediator, but not a moderator, in the relationship between EWB and ACS. 

## 4. Discussion

In recent years, the development of positive psychology has led to a notable increase in scientific interest in aspects related to positive psychological functioning. The important benefits of personal strengths and resources are solidly demonstrated in work environments [73] and, to a lesser extent, in academic settings [74]. However, the contribution that these personal resources can have on coping with academic demands during university studies has enjoyed scant attention to date, despite the high vulnerability to stress demonstrated by students in higher education [1,2]. 

This study is part of this incipient body of work analyzing the role played by the two personal resources of EWB and SE in adaptive coping with academic stress during university studies. The main contribution of this study is the analysis of the possible role of SE as a mediator and/or moderator variable of the relationship between EWB and the use of ACS. 

Regarding the study of mediation, consistent with our initial hypothesis, the results reveal that EWB has not only a significant and positive direct effect on ACS but also an indirect effect through SE. Thus, on the one hand, it has been confirmed that the greater the EWB experienced by students, the greater their use of highly functional coping strategies, such as positive reevaluation, seeking support, and planning. At the same time, has been demonstrated that SE is a positive significant predictor of adaptive coping with stress. These results, along the lines suggested by other articles [15,17,30], appear to indicate that both EWB and SE constitute valuable personal resources that directly favor adaptive coping with academic demands in the university phase. 

On the other hand, our results also demonstrate that the effect of EWB on the use of ACS is partially mediated by SE, which has a significant, positive, and large indirect effect. Some previous studies carried out among university students state that SE plays an important role in the stress process, mediating the relationship between personal resources such as positive emotions or mindfulness and adaptive coping with stress [39,40]. The data in this study allow us to deepen our understanding of this relationship, indicating that SE partially mediates the relationship between the most genuine and representative construct of human positive psychological functioning, which is EWB [14], and the use of ACS. Accordingly, a high level of development of students’ individual potential increases their belief in their own competence, which would notably increase their use of highly adaptive strategies in response to academic stressors (and vice versa).

The mediating role of SE would also be consistent with COR theory [35], in that it appears to confirm that those students who possess high personal resources (EWB) are more inclined to acquire and maintain new resources (SE), such that the accumulation of resources would bring important personal benefits (adaptive coping with academic problems). From this perspective, people who attain high levels of personal authenticity (i.e., EWB) and SE would possess psychological capital that would predispose them to face the present and future with expectations of success [75] and be more resistant to stress [76].

The second objective of this study sought to determine whether SE, in addition to this mediating role, also moderates the relationship between EWB and the use of ACS. Although diverse studies carried out in work settings highlight the important moderating role played by SE in preventing stress in conditions of low personal psychological adjustment [45,46]; our data does not confirm this assumption. Thus, contrary to what was hypothesized, we did not find a significant interaction between EWB and SE with regard to its effect on ACS. In this sense, it was observed that there is a greater use of these strategies when high EWB is accompanied by high SE. However, the use of these strategies decreases when the level of EWB is low, even when high SE is present.

SE constitutes a self-referencing belief that contributes greatly to the assessment of demands as challenges [23,28], thereby promoting the adoption of good stress coping mechanisms [25,29], in light of our results, its positive effect is attenuated in the absence of EWB. This finding is once again in line with what is indicated by COR theory [35], although only partially. This theory postulates that individuals who enjoy greater personal resources are in a situation of lesser vulnerability to stress than individuals with fewer resources [36]. Our results appear to confirm this assumption. However, this theory also contends that lack of personal resources (e.g., EWB) can be alleviated by other resources (e.g., SE), assertions that do not appear to support our findings. This unexpected result might perhaps be explained in light of social-cognitive theory and control theory. According to the social-cognitive theory [77,78], in some cases, specifically when individuals have to plan how to successfully cope with tasks in preparatory (e.g., learning) contexts (it should be noted that planning is one of the adaptive coping strategies considered in the present study), high levels of SE could negatively influence motivation to overcome challenges. It has been argued that high SE might lead students to feel sufficiently prepared, and therefore, less motivated to prepare further. In a similar vein, Vancouver and Kendall [79] used control theory [80] to state that high SE could lead students to estimate and allocate fewer resources (e.g., planning, and seeking support) for coping with academic demands. Therefore, both social-cognitive theory and control theory provide a reasonable explanation for the absence of a positive effect of SE on ACS when EWB is low. Under such circumstances, it could be possible that students with higher self-efficacy lack other personal resources (e.g., feelings of personal development, significantly desired personal goals, etc.) required to adaptively cope with academic problems. However, future studies should address this hypothetical explanation in a more rigorous manner.

In short, the fact that SE mediates but does not moderate the effect of EWB on ACS appears to indicate that SE is an important personal resource for producing a higher use of effective coping strategies among university students who enjoy high EWB. However, by itself, SE would be insufficient when EWB is low. Thus, it could be assumed that the tendency to effectively cope with academic stress increases significantly when both students’ EWB and SE are higher. This statement particularly seems to align with expectancy-value theory [81]. Conforming to this theory [82], the students’ persistence behavior in the face of difficulties is more likely to surface when it arises from the interaction between high expectancies—personal beliefs about one’s competence (i.e., SE)—and high personal values (i.e., the extent to which individuals feel the importance of doing well, try to attain important positive psychological consequences for themselves, or consider that a given task fits into their personal future plans) that are closely related to the notion of EWB. Meanwhile, it should be noted that the moderation analysis revealed that gender constitutes a significant moderating variable in the relationship between EWB and ACS. Thus, women and men use these strategies equally when EWB is high, but when it is low, the adoption of ACS is significantly higher among men. This finding appears to confirm the existence of gender differences in coping with academic stress in university studies [47,48]. In this sense, it seems that women tend to resort more to passive strategies such as rumination, self-blame or coping inhibition [83], while men tend to opt for some type of active strategy [84]. Based on this consideration, it can be speculated that, when high personal psychological resources are present, women, like men, tend to evaluate problems as less threatening, adopting highly adaptive strategies. However, the lack of these personal resources would increase women’s tendency to adopt passive strategies, while male students, perhaps due to their greater level of dispositional optimism [85], would preserve their tendency toward active coping even when experiencing low EWB. Future articles should analyze this assumption in greater depth. 

### 4.1. Educational and Health Implications

In our view, the results of this study contribute to a broadening of the spectrum of interventions geared toward preventing academic stress in university. Our data suggest that EWB, and specifically the dimensions comprising it (self-acceptance, environmental mastery, life purpose, personal growth), constitute a very valuable personal resource that favors adaptive coping in the face of academic stressors. This finding suggests the relevance of placing the focus of attention on the promotion of students’ full personal potential. Along these lines, so-called Positive Psychological Interventions may constitute an effective tool for favoring EWB in university students [86]. This initiative proposes an intervention focused on, among other aspects, personal strengths, with the objective of helping students to discover, cultivate, and utilize their personal values, motivations, resources and virtues appropriately. In this sense, past research [74] provide good examples about the benefits (in terms of SE, self-esteem, and perceived confidence for learning autonomously) of enhancing psychological character strengths in higher education.

Similarly, our results suggest the benefits of this type of intervention considering the important role played by SE, as this resource constitutes one of the links through which EWB is related to ACS. Consequently, future articles should analyze the specific changes that occur in students’ SE during interventions focused on the promotion of their EWB and how these changes positively affect strategies for coping with academic stress. 

### 4.2. Limitations of the Study and Areas for Future Research

While the results of this study contribute to the understanding of the relationship between EWB and adaptive coping with university stress and the role of SE in this relationship, the limitations of the study must be considered. First, the correlational nature of the research design makes it impossible to establish causal relationships between the variables analyzed. This issue should be addressed in the future through longitudinal research designs. Second, other variables that intervened in the results, whether as antecedents, mediators and/or modulators, were not considered in the study. Thus, future research should determine whether other psychological resources affect the use of ACS. Regarding these strategies, we should state as a third limitation the fact that this study only considered positive re-evaluation, seeking support and planning. While these three strategies constitute good examples of adaptive academic coping [9], the use of a more complete classification of strategies (both adaptive and maladaptive) would expand on our understanding of the role of EWB and SE in coping with academic stress. In this regard, a possible area for future research would seek to determine the extent to which EWB and SE have a protective effect (direct or indirect) on the use of dysfunctional coping strategies. 

A fourth limitation of this study lies in the use of self-report instruments as the exclusive data-gathering procedure. In this sense, a combination of methodologies that involve classroom observation, surveys and interviews with students would provide greater richness and rigorousness to the study of students’ psychological resources and their relationship to managing academic demands.

The characteristics of the sample utilized could also constitute a limitation of this study. Although it is rich from a quantitative point of view, all the participants come from the same university. To generalize our findings to the university student population as a whole, future research should attempt to replicate this study with students from other geographic and cultural contexts. Similarly, more research sampling techniques than those used in this study should be applied in the future.

## 5. Conclusions

The main contribution of this article relates to the joint effect of EWB and SE on adaptive coping with academic stress at university. Specifically, the role of SE as a mediator and moderator of the relationship between EWB and coping strategies was analyzed. Our results indicate that SE partially mediates this relationship but does not moderate it. Therefore, SE constitutes a relevant personal resource that favors adequate coping with academic stress, but it is insufficient by itself when the level of EWB of students is low. These findings may constitute an important contribution to the field of the prevention of academic stress, placing the focus on the promotion of EWB and SE as personal psychological resources of great value.

## Figures and Tables

**Table 1:** Descriptive statistics and Pearson correlation matrix.

	1	2	3	4
Gender	-			
2. EWB	−0.059 *	-		
3. SE	−0.255 **	0.532 **	-	
4. ACS	−0.061 *	0.551 **	0.575 **	-
*M*	1.69	3.93	3.35	3.21
*SD*	0.46	0.53	0.68	0.56
Asymmetry	−0.81	−0.67	0.02	−0.07
Kurtosis	−1.34	0.84	−0.52	−0.28

Note: Gender (Male = 1; Female = 2); EWB = Eudaimonic Well-Being; SE = Self-Efficacy; ACS = Adaptive Coping Strategies; * *p* < 0.05; ** *p* < 0.01.

**Table 2:** Results of the mediational analysis.

	Coefficient	SE	*t*	*p*	LLCI	ULCI
Dependent variable: SE						
EWB	0.673	0.028	23.705	0.000	0.617	0.728
Gender	−0.332	0.032	−10.287	0.000	−0.395	−0.268
Dependent variable: ACS						
SE	0.341	0.021	16.509	0.000	0.300	0.381
EWB	0.360	0.026	13.888	0.000	0.309	0.411
Gender	0.078	0.026	3.030	0.003	0.028	0.129
Dependent variable: ACS						
EWB	0.589	0.024	24.622	0.000	0.542	0.636
Gender	−0.035	0.027	−1.279	0.201	−0.088	0.019

Note: EWB = Eudaimonic Well-Being; SE = Self-Efficacy; ACS = Adaptive Coping Strategies; LLCI = lower level for confidence interval; ULCI = upper level for confidence interval; Gender (Male = 1; Female = 2).

**Table 3:** Results of the hierarchical regression analysis.

	Coefficients	Nonstandardized		Standardized		
Model		*B*	*SE*	*Beta*	*t*	*p*
	Constant	3.079	0.045		68.160	0.000
	Gender	0.076	0.026	0.062	2.940	0.003
1	EWB	0.232	0.081	0.216	2.843	0.005
	SE	0.339	0.021	0.411	16.451	0.000
	Gender × EWB	0.078	0.047	0.125	1.667	0.096
	Constant	3.070	0.045		67.605	0.000
	Gender	0.075	0.026	0.062	2.919	0.003
2	EWB	0.195	0.084	0.182	2.314	0.005
	SE	0.338	0.021	0.409	16.373	0.000
	Gender × EWB	0.103	0.049	0.167	2.112	0.035
	EWB × SE	0.051	0.030	0.037	1.688	0.092
Conditional adjustment between the two models						
Model	*SS_RES_*	*df_RES_*	*SS_RES_*	*df_RES_*	*F*	*p*
1	259.892	1398				
2	259.362	1397	0.530	1	2.855	0.091

Note: EWB = Eudaimonic Well-Being; SE = Self-Efficacy; ACS = Adaptive Coping Strategies. Gender (Male = 1; Female = 2).

## Supplementary Materials

Supplementary File 1
