# Supplementary material for: Eudaimonic Well-Being and Coping with Stress in University Students: The Mediating/Moderating Role of Self-Efficacy
Source: Int J Environ Res Public Health. 2018 Dec 25;16(1):48. doi: 10.3390/ijerph16010048 (PMC6339215; doi:10.3390/ijerph16010048)
Supplement: Supplementary File 1 [file ijerph-16-00048-s001.pdf]

Regulamento do Comité de Ética da Investigación e a Docencia da Universidade da Coruña (CEID-UDC)

Aprobado polo Consello de Goberno o 03/04/2018

Artigo 1. Comité de Ética da Investigación e a Docencia

O Comité de Ética da Investigación e a Docencia é un órgano colexiado, permanente, interdisciplinar, independente e de carácter consultivo que ten como finalidade avaliar e garantir a corrección dos aspectos éticos da investigación e a docencia e promover a integridade científica e académica.

Artigo 2. Ámbito de actuación

1. O CEID exerce as súas funcións respecto da investigación e a docencia desenvolvidas nos grupos, centros de investigación e centros propios da Universidade da Coruña ou nos que esta participe, e daqueles que estean adscritos a ela, así como respecto das actividades dos investigadores vinculados a calquera destes.

2. O CEID ocuparase das cuestións relacionadas coa integridade científica e académica dos estudantes, o persoal docente e investigador, o persoal de administración e servizos e/ou os cargos de dirección e xestión da UDC.

Artigo 3. Adscrición

O CEID está adscrito organicamente á Vicerreitoría de Política Científica, Investigación e Transferencia da UDC, que lle proporcionará o apoio económico, técnico e administrativo necesario para o seu bo funcionamento.

Artigo 4. Funcións

1. O CEID desempeñará as súas funcións con independencia, autonomía e imparcialidade.

2. Son funcións do CEID:

a) Informar sobre a corrección ética dos proxectos e traballos de investigación.
b) Asesorar os órganos competentes sobre os aspectos éticos relacionados coa docencia.
c) Garantir a integridade científica e académica.
d) Responder as consultas sobre cuestións éticas da investigación e a docencia.
e) Fomentar a integridade científica e académica e o respecto da ética da investigación e a docencia como criterios de calidade e de boa práctica profesional.
f) Elaborar informes, recomendacións, protocolos e códigos de boas prácticas.
g) Calquera outra relacionada cos seus fins que lle sexa expresamente encomendada pola UDC.

Artigo 5. Composición

1. A composición do CEID será interdisciplinar e plural, e procurarase garantir a presenza de todas as ramas de coñecemento: artes e humanidades, ciencias, ciencias da saúde, ciencias sociais e xurídicas, e enxeñaría e arquitectura.

Firmante: Universidade da Coruña - Q6550005J   URL de validación: https://sede.udc.gal/services/documents_validation   CSV: A6U11HTMQBGM1UANH343IB5F
Este documento es copia auténtica de un documento custodiado por la Universidade da Coruña. Fecha de creación: 05/04/2018 14:02:17 Página 1 de un total de 3 página(s).

2. O CEID contará cun mínimo de cinco e un máximo de once vogais pertencentes ao persoal docente e investigador da UDC**.** Polo menos un deles debe ter formación xurídica.

3. Garantirase a presenza equilibrada de mulleres e homes.

Artigo 6. Estrutura orgánica

1. O CEID consta dunha presidencia, unha secretaría e vogalías.

2. A persoa titular da presidencia será elixida polos vogais do CEID entre eles en sesión plenaria por maioría simple.

Correspóndelle á persoa que ocupa a presidencia ordenar a convocatoria, coordinar e dirixir as sesións do CEID, e representalo institucionalmente.

3. A secretaría do CEID corresponderalle a un membro do persoal de administración e servizos da UDC.

Correspóndelle á persoa encargada da secretaría a xestión e a organización administrativa do CEID.

Artigo 7. Pertenza e exercicio

1. A condición de membro do CEID é persoal, intransferible e indelegable.

2. O mandato dos membros será de catro anos, renovable por un único período de igual duración.

3. Os membros serán nomeados e cesados polo/o reitor/a por proposta da Vicerreitoría de Política Científica, Investigación e Transferencia, que informarán o Consello de Goberno da UDC. O nomeamento e o cesamento serán públicos.

4. A pertenza ao CEID finalizará por renuncia expresa, terminación do mandato, perda de vinculación coa UDC ou cesamento por incumprimento reiterado das obrigas propias do cargo.

5. Os membros do CEID deixarán constancia expresa, nun rexistro creado para ese efecto no ámbito da Vicerreitoría de Política Científica, Investigación e Transferencia, das actividades e as circunstancias capaces de xerar un conflito de intereses, para os efectos que procedan.

6. Os membros do CEID teñen deber de confidencialidade sobre os asuntos que coñeceren con ocasión do seu cargo e sobre os procesos de deliberación e votación das súas reunións.

7. A participación no CEID será recoñecida a través da compensación ou redución da carga docente.

Artigo 8. Funcionamento

1. O CEID actuará a través do pleno e as comisións.

2. O pleno está composto pola persoa que exerce a presidencia, a persoa que exerce a secretaría e os/as vogais. O pleno reunirase con periodicidade mensual, excepto no mes de agosto.

3. O pleno do CEID pode crear comisións para o estudo e a avaliación de cuestións específicas. O acordo de creación indicará a súa composición, duración, funcións, obxectivos e prazos. As comisións estarán compostas polo menos por tres vogais e a persoa que desempeña a secretaría.

4. Os acordos das comisións deberán ser ratificados polo pleno co voto favorable da maioría dos membros do CEID.

5. O CEID poderá nomear consultores externos expertos nunha área de especialización concreta, que actuarán como asesores con voz, pero sen voto.

Firmante: Universidade da Coruña - Q6550005J URL de validación: https://sede.udc.gal/services/documents_validation CSV: A6U11HTMQBGM1UANH343IB5F
Este documento es copia auténtica de un documento custodiado por la Universidade da Coruña. Fecha de creación: 05/04/2018 14:02:17 Página 2 de un total de 3 página(s).

Disposición adicional. Procedemento de reforma

A modificación do presente Regulamento correspóndelle ao Consello de Goberno da UDC.

A iniciativa para a modificación corresponde ao Consello de Goberno e tamén, cando o decida así por maioría absoluta, á Comisión de Investigación e ao CEID.

Disposición derrogatoria. Derrogación de normativa

Fica derrogada toda a normativa propia da UDC de rango igual ou inferior a este regulamento que contradiga ou se opoña ao que nel se dispón.

Fica derrogado expresamente o Regulamento do Comité de Ética da Universidade da Coruña aprobado polo Consello de Goberno o 23/07/2008.

Disposición final. Entrada en vigor

O presente Regulamento entrará en vigor ao día seguinte da súa publicación no Taboleiro Electrónico Oficial da UDC.

A Coruña, 3 de abril de de 2018

Firmante: Universidade da Coruña - Q6550005J URL de validación: https://sede.udc.gal/services/documents_validation CSV: A6U11HTMQBGM1UANH343IB5F
Este documento es copia auténtica de un documento custodiado por la Universidade da Coruña. Fecha de creación: 05/04/2018 14:02:17 Página 3 de un total de 3 página(s).
